# COVID-19 Workplace Mitigation Strategies and Employee Leave Policies Implemented during the Height of the Pandemic, United States, Fall 2020 and 2021

**DOI:** 10.3390/ijerph20042894

**Published:** 2023-02-07

**Authors:** Rebecca J. Guerin, John P. Barile, Matthew R. Groenewold, Hannah L. Free, Andrea H. Okun

**Affiliations:** 1 Division of Science Integration, National Institute for Occupational Safety and Health, Centers for Disease Control and Prevention, Cincinnati, OH 45226, USA; 2 College of Social Sciences, Department of Psychology, University of Hawai‘i at Mānoa, Honolulu, HI 96822, USA; 3 Division of Field Studies and Engineering, National Institute for Occupational Safety and Health, Centers for Disease Control and Prevention, Cincinnati, OH 45226, USA

**Keywords:** COVID-19, workplace mitigation strategies, prevention strategies, workplace safety and health, occupational safety and health, small business, small enterprise, essential industries

## Abstract

COVID-19 workplace mitigation strategies implemented within US businesses have been effective at preventing disease and protecting workers, but the extent of their use is not well understood. We examined reported COVID-19 workplace mitigation strategies by business size, geographic region, and industry using internet panel survey data from US adult respondents working full- or part-time outside the home (fall 2020, *N* = 1168) andfull- or part-time, inside or outside the home (fall 2021, *N* = 1778). We used chi-square tests to assess the differences in the strategies used (e.g., masking and COVID-19 screening) and ANOVA tests to examine the group differences on a mitigation strategies summative score. Fewer COVID-19 mitigation strategies were reported by respondents in fall 2021 (compared to fall 2020) across businesses of different sizes and regions. The participants in microbusinesses (1–10 employees) reported significantly (*p* < 0.05) lower mitigation scores than all other business sizes, and the respondents in these businesses were significantly less likely (*p* < 0.05) to have paid leave than those in enterprises with >10 employees. The healthcare and education sectors had the highest reported mean score of COVID-19 workplace mitigation strategies. Small and essential businesses are critical to the US economy. Insight is needed on their use of mitigation strategies to protect workers during the current and future pandemics.

## 1. Introduction

Some workplaces have been high-risk environments for COVID-19 outbreaks and transmission [1,2]. While workplace closures likely reduced COVID-19 transmission early in the pandemic [3,4], those performing essential functions remained open and implemented mitigation strategies, such as face masking, physical distancing, and hand hygiene, to prevent the spread of COVID-19 [3,5]. Research on reducing influenza transmission in workplaces [6], from the H1N1 pandemic [7,8] and the COVID-19 pandemic [1], demonstrates the effectiveness of various workplace mitigation strategies, such as physical distancing and employer leave policies, at preventing disease spread, especially when the strategies are layered [1,6]. Early in the pandemic, the US Centers for Disease Control and Prevention (CDC) released and continuously updated guidance for businesses and employers to help prevent workplace exposures to COVID-19 [9]. The pandemic disproportionately affected workers in certain essential industries, including healthcare, the food supply chain, and public safety [10,11,12,13]. The US Occupational Safety and Health Administration (OSHA) also released (and updated) guidance for preparing various industries for operation during the pandemic [14,15]. This guidance provided recommended general mitigation strategies for reducing the transmission of COVID-19 in workplaces, such as requiring physical distancing, mask wearing, and practicing routine cleaning and disinfection [9,14]. Specific worker protection guidance was also issued for healthcare settings [16,17].

Available research provides a limited perspective on the extent to which COVID-19 prevention measures were implemented early in the pandemic. A US national survey from June 2020 demonstrates that among nonhealthcare workers, fewer than one-half of the respondents reported the use of hazard controls, such as gloves or a respirator, to prevent COVID-19, and slightly more than one-half reported the required use. Voluntary use was approximately double among workers whose employers provided hazard controls than among those whose employers did not [5]. A report from the US National Safety Council (NSC) that surveyed more than 300 safety and health professionals working in business with at least 250 employees (in summer 2020) indicated that across nearly all industries represented in the sample, organizations implemented multiple COVID-19 mitigation strategies. These included investing in hand washing and hand sanitization stations; implementing procedures to increase the frequency of cleaning and sanitization; providing proper PPE; facilitating physical distancing by allowing for remote work arrangements for nonessential workers; and installing signage [18]. Research from Japan conducted in 2020 using national online panel data indicated that fewer workplace prevention measures were implemented in smaller companies and in the retail, wholesale, and transportation industries [19] compared to other business sizes and industries. A study from Iran with 255 businesses reported that large organizations (>100 employees) implemented significantly more preventative actions to reduce COVID-19 transmission than smaller enterprises, with businesses in the healthcare sector having implemented more COVID-19 mitigation strategies compared to other industries represented in the sample [20]. Little is currently known regarding the implementation of COVID-19 workplace mitigation strategies during the height of the pandemic in the United States; how implementation differs by important enterprise characteristics, such as size and location; and how businesses changed their mitigation strategies as the pandemic progressed.

The purpose of the current study was to address this research gap by estimating the reported prevalence of COVID-19 workplace mitigation strategies and leave policies implemented within US workplaces by business size, geographic region, and industry in fall 2020 and 2021. This analysis focuses on general business and workplace mitigation strategies and not on specific guidance for protecting employees providing direct patient care within healthcare settings [16]. Given the importance of workplace mitigation strategies and leave policies for preventing disease transmission [7,8,21], the current study adds to the literature by exploring the implementation of COVID-19 workplace mitigation strategies and leave policies as reported by workers in two nationally representative surveys. This study’s results may inform current and future responses to public health emergencies.

## 2. Materials and Methods

### 2.1. Participants and Procedure

The data were collected by Porter Novelli Services for their 2020 (24 September 2020–10 October 2020) and 2021 (24 September 2021–7 October 2021) FallStyles surveys [22], fielded via an online, opt-in panel [23] conducted in English and weighted to US adult population statistics from the 2019 US Current Population Survey [24]. Panel members were randomly recruited by mail using probability-based sampling by address from a pool of approximately 60,000 eligible respondents. The 2020 FallStyles survey was sent to a sample of 4548 panelists aged 18 years or older who participated in the 2020 SpringStyles survey (March–April 2020). The 2021 FallStyles survey was sent to a sample of 4510 panelists who participated in the 2021 SpringStyles survey. Non-responders received three email reminders. A total of 3625 adults completed the survey in 2020 (response rate of 79.7%), and a total of 3553 adults completed the survey in 2021 (response rate of 78.8%). The surveys took approximately 33 min (fall 2020) and 37 min (fall 2021) to complete, and participants earned cash-equivalent reward points worth approximately $5–$10.

The survey proportions were weighted for sex (male/female), age (18 to 24 years, 25 to 34 years, 35 to 44 years, 45 to 54 years, 55 to 64 years, and ≥65 years), pre-pandemic household income (21 categories, range: (<$5000, ≥$250,000)), race and ethnicity (White, Non-Hispanic persons; Black, Non-Hispanic persons; all other races, Non-Hispanic persons; more than one race, Non-Hispanic persons; and Hispanic/Latino persons), household size (one, two, three, four, and more than or equal to five), educational attainment (less than high school; high school; some college; and Bachelor’s degree and higher) census region (Northeast, Midwest, South, and West), and metro status (as defined by the US Office of Management and Budget (OMB) Core-Based Statistical Areas) (non-metro and metro).

The sample for the current analysis included those respondents indicating that they were working as a paid employee or were self-employed. Those indicating they were not working for various reasons (retired, disability, temporary layoff, looking for work, and other) were excluded from the sample. No questions regarding the job role/s of the respondents were included on the surveys. For our analysis, in fall 2020, we included only those respondents who worked outside the home (either full- or part-time) in the four weeks preceding the survey. In fall 2021, the analysis included all those working either full- or part-time at the time of the survey. Therefore, respondents could have been working inside or outside the home, as many individuals were working blended schedules by the fall of 2021. The inclusion of respondents with complete data on the variables of interest resulted in a fall 2020 sample of *N* = 1168 (*N* = 1070 unweighted) and a fall 2021 sample of *N* = 1778 (*N* = 1755 unweighted).

### 2.2. Measures

#### 2.2.1. Outcomes Variables

The outcome variables included the use (yes or no) of selected COVID-19 workplace mitigation strategies and types of employee leave policies, as reported by respondents in the sample. These mitigation strategies were based on CDC and OSHA guidance for businesses and employers to help prevent workplace exposures to COVID-19 [9,14,15]. For fall 2020, the respondents were asked to complete the mitigation questions, which included: “‘Has your primary employer done any of the following to prevent the spread of COVID-19?’ Select all that apply.” Response choices included: (1) implemented safe distancing (6 feet or more) between employees and/or customers; (2) provided respirators (like N95s) to employees; (3) required employees to wear a mask; (4) required customers/clients to wear masks; (5) screened employees (asking about symptoms, taking temperatures, etc.); (6) screened customers/clients for COVID-19 symptoms; (7) reassigned workers at increased risk for severe illness (older, underlying conditions); (8) put up physical barriers such as partitions or sneeze guards; (9) used enhanced cleaning/disinfection procedures; (10) tested employees for the virus that causes COVID-19; (11) provided COVID-19 prevention training to employees; (12) limited the number of customers in the establishment at one time; (13) provided hand sanitizer or handwashing supplies (such as soap and drying materials); (14) posted signs about safe practices (like social distancing, masks, handwashing); (15) none of these actions were taken to prevent the spread of COVID-19. In fall 2021, due to the fact of space limitations on the survey, we removed two items (provided respirators (such as N95s) to employees and posted signs about safe practices) and combined two items (screened employees for COVID-19 symptoms and screened customers/clients for COVID-19 symptoms). We replaced these items with three additional COVID-19 mitigation strategies to reflect the changing nature of the pandemic over the preceding year, including: (1) required employees to be vaccinated against COVID-19; (2) used enhanced ventilation at the worksite; (3) moved to remote working (such as teleworking).

To allow for comparisons across waves, a summative score (ranging from 0 to 11) was created using the total number of COVID-19 workplace mitigation strategies reported by each respondent for those mitigation strategies that were included on both the 2020 and 2021 surveys. All mitigation strategies were treated equally in the calculation of the summative score. Mitigation strategies included on only one survey were examined separately.

To assess employee leave policies in fall 2020, respondents were asked: “‘At your primary job, does your employer offer any type of leave in response to COVID-19?’ Select all that apply.” Response choices included: (1) paid leave if I have COVID-19 symptoms; (2) paid leave if I test positive for COVID-19; (3) unpaid leave—time off without pay; (4) no leave–I cannot take off; (5) do not know. In fall 2021, this question was expanded to provide the following response choices (select all that apply): (1) general paid annual/vacation leave; (2) general paid sick leave; (3) paid leave only for COVID-19 symptoms; (4) paid leave only for positive COVID-19 test; (5) unpaid leave—time off without pay; (6) no leave—I cannot take off; (7) do not know. Between year comparisons were not conducted for this question due to the fact of these changes.

#### 2.2.2. Independent Variables

The respondents provided information on the following items that were used in the analysis as independent variables: employer’s business size by number of employees (1–10 (i.e., “microbusinesses”), 11–50, 51–100, and >100); geographic region (Northeast, Midwest, South, West); and employer’s industry (2-digit NAICS industries) (The North American Industry Classification System (NAICS) is the standard used by federal statistical agencies in classifying business establishments for the purpose of collecting, analyzing, and publishing statistical data related to the US business economy (https://www.census.gov/naics, accessed on 11 November 2022), for industry sectors with ≥100 respondents prior to eliminating those who did not receive the mitigation questions, including manufacturing; retail trade; information services; finance and insurance; professional, scientific, and technical services; educational services; healthcare and social assistance; and all “other” industries).

### 2.3. Statistical Analyses

Chi-square tests for significant proportional differences in COVID-19 mitigation strategies and employee leave policies were conducted by business size, geographic region, and industry. Analysis of variance (ANOVA) tests were conducted to explore group differences on a summative workplace mitigation strategy score (ranging from 0 to 11, representing the total number of COVID-19 workplace mitigation strategies reported by each respondent) by industry, business size, and geographic region. Only those mitigation strategies that were included on both survey waves were included in the summative score so that comparisons could be made across waves. Mitigation strategies included on only one survey were assessed separately. Tukey post hoc tests were conducted to identify statistically significant differences among the groups. SPSS (version 28; SPSS IBM Inc., Armonk, NY, USA) was used to conduct all analyses. The majority of missing data in 2020 (9.09%) and in 2021 (8.92%) resulted from the participants not knowing the size of their company/business. 

## 3. Results

### 3.1. Descriptive Statistics

The region representing the largest proportion of survey respondents was the South in both fall 2020 (36.4%) and 2021 (35.9%). The respondents working in larger businesses with >100 employees comprised 41.8% and 51.7% of the sample in fall 2020 and 2021, respectively, while those working in microbusinesses with ≤10 employees made up 22.0% (2020) and 20.2% of the sample (2021). The respondents represented a wide range of industries including healthcare and social assistance, education, manufacturing, and retail trade (See Table 1 for descriptive statistics).

### 3.2. Reported Workplace Mitigation Strategies

**Figure 1:**
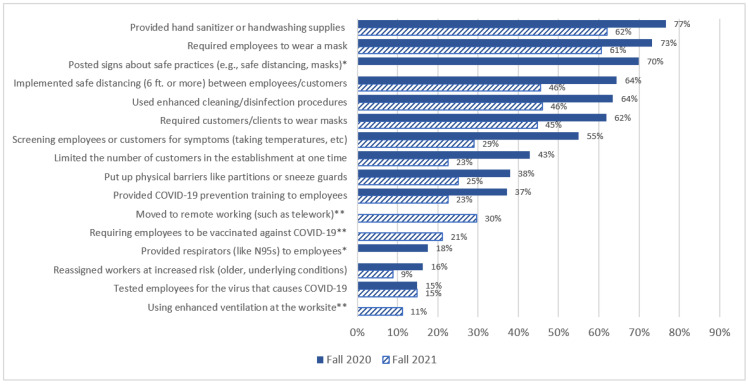
Respondent reported COVID-19 workplace mitigation strategies, fall 2020 and fall 2021. * Only asked in fall 2020; ** only asked in fall 2021.

In fall 2020, the five most common mitigation strategies reported were providing hand sanitizer or handwashing supplies (77%), requiring employees to wear a mask (73%), posted signs about safe practices (70%), safe distancing (64%), and using enhanced cleaning (64%) (Figure 1). These were also among the most commonly reported mitigation strategies in fall 2021: providing hand sanitizer or handwashing supplies (62%), requiring employees to wear a mask (61%), safe distancing (46%), and using enhanced cleaning (46%) (Figure 1). Of the mitigation strategies that were included on both the 2020 and 2021 surveys, tested employees for the virus that causes COVID-19 and reassigned workers at increased risk (for COVID-19) were the least frequently reported.

**Fall 2020 Mitigation Strategies**. Chi-square analyses revealed statistically significant differences by business size for each of the COVID-19 workplace mitigation strategies assessed (see Table S1 for full results). Specifically, participants who worked in microbusinesses (1–10 employees) and smaller businesses with 11–50 employees were significantly (*p* < 0.05) less likely than those employed in the largest businesses size category (>100 employees) to report implementation for 13 of the 14 assessed COVID-19 mitigation strategies. The exception was, “Limited the number of customers in the establishment at one time”, which was statistically significantly different (*p* < 0.05) only for the smaller (11–50 employees) versus the largest (>100 employees) businesses. When microbusinesses were compared to the business size categories with 11–50 and 51–100 employees, the participants who worked in microbusinesses were less likely to report safe distancing; requiring employees to wear a mask; screening employees for symptoms; and posting signs about safe practices. Smaller businesses with 51–100 employees were also significantly (*p* < 0.05) less likely than those employed by larger businesses (with >100 employees) to report safe distancing; requiring employees to wear a mask; putting up physical barriers; using enhanced cleaning/disinfecting procedures; testing employees for COVID-19; providing hand sanitizer or handwashing supplies; and posting signs about safe practices. The only statistically significant difference in the reported workplace mitigation strategies between the two middle-sized businesses categories (11–50 and 51–100 employees) was identified for the reassignment of workers at increased risk for severe illness.

The fall 2020 data (see Table S1) suggest limited employer provision of respirators (such as N95s) to employees (ranging from 8% for businesses with ≤10 employees to 26% for businesses with >100 employees). The respondents working in businesses with >100 employees were statistically significantly (*p* < 0.05) more likely to report employer provision of respirators to employees as compared to businesses with 50 or fewer employees.

**Fall 2021 COVID-19 Mitigation Strategies.** The differences in mitigation strategies by business size in fall 2021 were similar to those observed in fall 2020 (Table S1). In one departure from the fall 2020 results, the largest (>100 employees) business size category was significantly more likely to require customers/clients to wear masks (*p* < 0.05) compared to the three smaller sized business categories (≤100 employees). The fall 2021 data (see Table S1) suggest limited implementation of enhanced ventilation for COVID-19 (6% for businesses with ≤10 employees compared to 14% for businesses with >50 employees). The respondents working in businesses with >100 employees were statistically significantly (*p* < 0.05) more likely to report moving to telework (43%) as compared to businesses with 51–100 employees (25%), 11–50 employees (16%), or those with 10 or fewer employees (9%). Finally, given that COVID-19 vaccines were available in 2021, a question regarding a vaccine requirement for employment was added to the fall 2021 survey. Again, the respondents working for a business with >100 employees were statistically significantly (*p* < 0.05) more likely to report a vaccine requirement (28%) compared to those in smaller sized businesses (11–50 employees, 17%; 1–10 employees, 8%).

**Figure 2:**
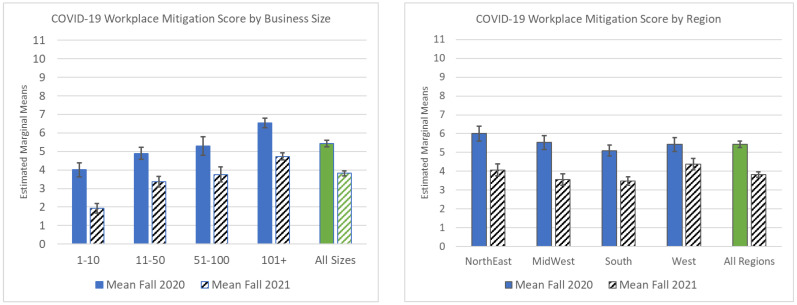
Respondent reported COVID-19 mitigation scores by business size (number of employees) and US region, fall 2020 and fall 2021.

**COVID-19 Mitigation Summary Scores.** The mitigation scores for both 2020 and 2021 were found to be normally distributed. In 2020, the summary scores had a mean of 5.43 and a median of 6.00, with a minimal kurtosis (−0.94) and skewness (−0.24). A 4 × 4 between-subjects factorial ANOVA used to determine whether the mitigation mean summative scores differed by business size (four levels) and geographic region (four regions) for fall 2020 and fall 2021 revealed that fewer COVID-19 mitigation strategies were reported in fall 2021 (compared to fall 2020) across businesses of different size and region (Figure 2). For fall 2020, when assessing the mitigation scores by geographic region within the four business size categories, there was a main effect for both region (F3,15 = 6.27; *p* < 0.001) and business size (F3,15 = 44.46; *p* < 0.001) on mitigation scores but no evidence of an interaction between business size by region (F9,15 = 1.32; *p* = 0.219) (data not shown). The respondents in the Northeast reported a significantly higher mean number of COVID-19 mitigation strategies (*p* < 0.001) than those in the South. No other statistically significant differences by region were observed. For fall 2021, when assessing the mitigation scores by geographic region within the four business size categories, there was a main effect for region (F3,15 = 3.34; *p* = 0.019) and business size (F3,15 = 97.97; *p* < 0.001) but again no evidence of an interaction between business size by region on mitigation scores (F9,15 = 1.54; *p* = 0.128) (data not shown). Figure 2 displays the differences in the mean COVID-19 mitigation scores by business size and region.

**Figure 3:**
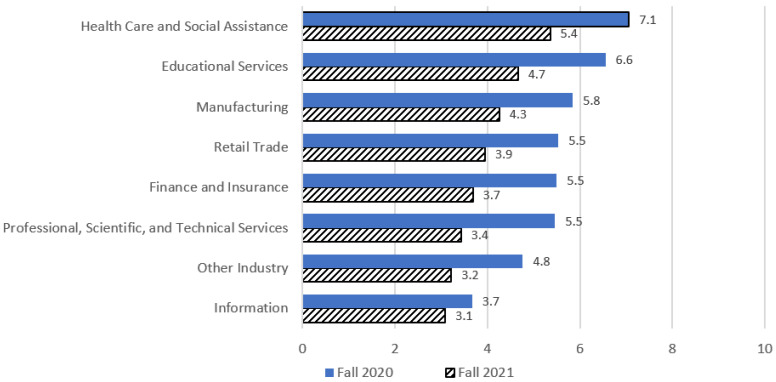
Respondent reported COVID-19 workplace mitigation scores (estimated marginal means, rounded to the nearest tenth) by industry, fall 2020 and fall 2021.

In 2021, the summary scores had a mean of 3.45 and a median of 3.00, with a minimal kurtosis (−0.73) and skewness (0.54). The results of a between-subjects ANOVA revealed significant differences in the mean mitigation scores between industries for fall 2020 and fall 2021 (graphically displayed in Figure 3). For fall 2020, the respondents who worked in the healthcare sector reported the highest mean number ($\bar{x}$ = 7.06, 95% CI: 6.58, 7.54) of COVID-19 mitigation strategies implemented in workplaces, which was statistically significantly higher (*p* < 0.05) than all other industry sectors with the exception of education (*p* = 0.866). The respondents who worked in the education sector reported the second highest mean number ($\bar{x}$ = 6.55; 95% CI: 6.01, 7.09) of COVID-19 mitigation strategies implemented in workplaces, which was statistically significantly higher than the industry categories of information (*p* = 0.009) and other industries (*p* < 0.001). The information industry reported the lowest mean number of COVID-19 mitigation strategies ($\bar{x}$ = 3.67; 95% CI: 2.17, 5.17). Similar results were observed for fall 2021, when the respondents who worked in the healthcare sector again reported the highest mean number ($\bar{x}$ = 5.36, 95% CI: 4.97, 5.70) of COVID-19 mitigation strategies implemented in workplaces, which was statistically significantly higher (*p* < 0.01) than all other industry sectors, except education (*p* = 0.254). The respondents who worked in the education sector reported the second highest mean number ($\bar{x}$ = 4.66; 95% CI: 4.23, 5.09) of COVID-19 mitigation strategies implemented in workplaces, which was statistically significantly higher than the industry categories professional/scientific/technical services (*p* < 0.001), information (*p* = 0.004), and other industries (*p* < 0.001). The information industry sector again reported the lowest mean number of COVID-19 mitigation strategies ($\bar{x}$ = 3.09; 95% CI: 2.39, 3.78). Figure 3 displays the differences in the mean COVID-19 mitigation by industry.

**Leave Policies.** For respondents working outside the home in fall 2020, <20% of employees in microbusinesses (1–10 employees) reported having paid leave if they had COVID-19 symptoms (17%) or if they tested positive for COVID-19 (18%) (Table 2). Among those employed in businesses with 11–50 employees, 21% reported having paid leave if they had COVID-19 symptoms and 26% if they tested positive for COVID-19. In the largest two business size categories, over 40% of employees reported paid leave if they had COVID-19 symptoms or if they tested positive for COVID-19, a result that was statistically significantly different (*p* < 0.05) when compared to the 1–10 and 11–50 business size categories. The employees in the business size categories with ≤50 employees were statistically significantly more likely (*p* < 0.05) to report having no leave compared to employees in business with >100 workers. In fall 2021, the respondents were asked if they had paid leave specific only to having symptoms of COVID-19 or if they tested positive for COVID-19. Only 6% of respondents in microbusinesses reported paid leave for COVID-19 symptoms or for a positive test, and up to 19% reported paid leave for COVID-19 symptoms and 25% for a positive COVID-19 test for larger business (>100 employees) (Table 2). The respondents in microbusinesses reported being statistically significantly less likely (*p* < 0.05) to have either general paid annual/vacation leave or paid sick leave and more likely to have no paid leave than those respondents in the sample in enterprises with >10 employees. The percentage of the respondents indicating general paid annual/vacation leave ranged from 35% for microbusinesses to 87% for businesses with >100 employees, and the percentage indicating general paid sick leave ranged from 27% for microbusinesses to 73% for businesses with >100 employees (Table 2). Tables S2 and S3 provide the distribution by industry for each of the individual COVID-19 mitigation strategies and leave polies and complete results of the significance tests performed.

## 4. Discussion

During the study period, the sample respondents indicated businesses of all sizes implemented a number of COVID-19 mitigation strategies in their workplaces. In 2020, our analysis indicates that the five most commonly reported mitigation strategies included provided hand sanitizer; required employees to wear a mask; posted signs about safe practices; implemented safe distancing; and used enhanced cleaning. When considering larger businesses with >100 employees, our results are consistent with those from data collected by NSC in July 2020 from businesses with ≥250 employees [18]. Overall, our data indicate that fewer COVID-19 mitigation strategies were reported in fall 2021 (compared to fall 2020) across businesses of different sizes and regions. In both the fall 2020 and fall 2021 surveys, the summative score for the 11 mitigation strategies assessed indicates that microbusinesses (1–10 employees) had significantly lower mitigation scores than all other business size categories, while the largest business size category (>100 employees) had significantly higher mitigation scores than all other business size categories assessed. The fall 2021 survey also revealed that the respondents in businesses with >10 employees were significantly (*p* < 0.05) more likely to report a requirement that employees be vaccinated against COVID-19 compared to those in business with ≤10 employees. The respondents in businesses with >50 employees were significantly (*p* < 0.05) more likely to report that their company used enhanced ventilation compared to the respondents in businesses employing ≤50 people. These results are consistent with research from Japan [19] and Iran [20] in the early stages of the pandemic demonstrating that the number of reported workplace mitigation measures that were implemented was generally lower for smaller companies. Even before the COVID-19 pandemic, research indicates that smaller businesses provided fewer health and safety programs and fewer benefits for workers when compared with larger enterprises [25,26,27,28,29,30]. Perceived barriers to small businesses implementing occupational safety and health (OSH) measures include a lack of dedicated safety and health staff, insufficient safety and health resources, challenges in identifying workplace hazards [27,28], and variation/diversity in terms of business age, structure, management, and culture [29]. According to the US Bureau of Labor Statistics (BLS), businesses with <10 employees represent approximately 76% of US private sector firms [31] and 11% of the US labor force [32]. Given the importance of small businesses to the US economy, it is critical that they have the resources and capacity to implement mitigation strategies to protect their employees. More implementation research is needed [33,34] to shed light on the barriers and facilitators to small business employer’s adoption of various mitigation strategies to protect workers, including cost and resource constraints [25,26,27,28,29,30]. Moreover, future research should explore employee mental health and well-being impacts and implications related to employers’ implementation of robust disease prevention measures during public health emergencies [25].

When assessing the mitigation scores by geographic region within the four business size categories, there was a main effect for region. However, there was no evidence of an interaction between business size and region on the mitigation scores. Further research with a larger sample of respondents would be useful in further probing these associations. Not surprisingly, employees in the healthcare sector reported the highest mean number of COVID-19 mitigation strategies implemented in workplaces, and with the exception of the of education sector, was statistically significantly higher than all other industry categories assessed. These results are consistent with research from Iran conducted early in the pandemic reporting that businesses in the healthcare sector implemented more COVID-19 mitigation strategies compared to other industries represented in the sample [20]. Because of the high potential for exposure to COVID-19 in workplaces in this industry, extensive guidance on mitigation strategies to prevent the spread of disease was issued by federal agencies early in the pandemic and updated regularly [16,17]. Our data indicate that employees in information services reported the lowest number of COVID-19 mitigation strategies implemented in their workplaces. Research conducted in Japan early in the pandemic using national online panel data to explore workplace COVID-19 prevention measures indicated that among the industrial sectors assessed, fewer workplace prevention measures were implemented in the retail, wholesale, and transportation industries [19], even though these industries are “customer facing”. Research is needed to identify and tailor outreach to businesses within industries that may better protect their employees through enhanced provision of (layered) mitigation strategies [1,21].

Our research demonstrates that employer leave policies are not uniformly implemented across businesses of varying sizes, and substantial disparities exist. When examining reported workplace leave provided to respondents in the fall of 2020, the survey participants who worked outside the home in micro- and smaller businesses (1–50 employees) were statistically significantly (*p* < 0.05) less likely to report having paid leave if they experienced symptoms of or tested positive for COVID-19, compared to those in business with >50 employees. A limitation of our analysis in fall 2020 was that the question was worded to ask specifically about leave related to COVID-19 (“At your primary job, does your employer offer any type of leave in response to COVID-19?”). Some respondents may have been reporting only on whether their employer had instituted COVID-specific leave policies and not on whether they had access to general leave that could be used for COVID-19 symptoms/illness. We therefore changed the question in fall 2021 to ask about employer provision of general paid annual and sick leave in addition to leave specific to only having symptoms of COVID-19 or a positive COVID-19 test. We were not able to make cross-year comparisons due to the fact of this change. Using the fall 2021 data, the respondents in microbusinesses reported being statistically significantly less likely (*p* < 0.05) to have either general paid annual/vacation leave or paid sick leave and more likely to have no paid leave than those respondents in enterprises with >10 employees. Overall, only 27% of those in businesses with 1–10 employees and 55% of respondents in businesses with 11–50 employees reported having paid sick leave. Estimates from the US Bureau of Labor Statistics from March 2021 indicated that 68% of workers in private industry establishments (and 86% of state and local government employees) with <50 employees had access to paid sick leave [35]. However, an important caveat is that our data were not sampled for industry or business size. The BLS data also revealed that paid sick leave was available to 75% of all private industry workers in March 2021, ranging from 59% of workers in service jobs to 93% of workers in management, professional, and related occupations [36]. Given that access to sick leave has been shown to be an important factor in reducing the spread of infectious illnesses in workplaces [7,37,38], further research is needed to understand the impact of generous employer leave policies in promoting employee health and well-being [39] and in preventing the spread of disease in workplaces in this and future pandemics.

The findings in this report are subject to several limitations. First, the data were self-reported and there is potential for recall and response bias. Second, the two waves of data are not directly comparable because in fall 2020 the respondents were limited to those working outside the home while in 2021, as businesses reopened, the respondents could have been working inside or outside the home. Third, the list of mitigation strategies was not exhaustive, and the response options varied slightly between survey waves to account for the changing nature of the pandemic between survey years. Fourth, our survey did not account for the differences in the need for various mitigation strategies within industries. For example, not all industries require the same level of in-person interaction with customers and coworkers. Thus, the need for mask wearing, physical distancing, and other mitigation strategies is not uniform. Fifth, we did not distinguish between employers and employees in our survey and, thus, the responses could have varied depending on the respondent’s role, and the extent of their knowledge regarding the mitigation strategies in their particular workplace. In the same vein, owner–operators and those who are self-employed may also have substantially different experiences than those employed by others. Sixth, as previously described, slight changes to the survey questions limited cross-year comparisons in some cases. Finally, internet surveys vary in methodology and quality, and lower response rates from diverse socioeconomic and racial/ethnic minority groups are common [40]. Future research would benefit from a longitudinal design that measures outcomes at different time points with the same respondents and/or focused on employers in and owners of small and microbusinesses.

## 5. Conclusions

Workplaces have been shown to be high-risk environments for COVID-19 transmission, and several COVID-19 mitigation strategies have been demonstrated to be effective in preventing the spread of disease and protecting workers. However, their implementation in US businesses during the height of the pandemic has not been well understood. The current research addressed this critical research gap by revealing important differences in the use of mitigation strategies by business size, industry, and geographic region. Future research should examine how differences in the implementation of COVID-19 workplace mitigation strategies may have had an impact on the health and well-being of employees and how public health agencies can enhance employers’ efficient use of these prevention strategies to protect worker health and well-being in current and future public health emergencies.

## Figures and Tables

**Table 1:** Business Characteristics Reported by Survey Respondents in Fall 2020 and Fall 2021.

	Fall 2020 Sample (Weighted *N* = 1168) *		Fall 2021 Sample (Weighted *N* = 1778) *	
	*N*	%	*N*	%
Region				
Northeast	212	18.2	329	18.5
Midwest	250	21.4	384	21.6
South	425	36.4	639	35.9
West	280	24.0	425	23.9
Business Size (Number of employees)				
1–10	257	22.0	359	20.2
11–50	271	23.2	334	18.8
51–100	152	13.0	166	9.4
>100	488	41.8	919	51.7
Industry				
Manufacturing	128	11.0	145	8.2
Retail Trade	131	11.2	186	10.4
Information	15	1.3	62	3.5
Finance and Insurance	43	3.6	130	7.3
Professional, Scientific, and Technical Services	76	6.5	196	11.0
Educational Services	108	9.3	165	9.3
Healthcare and Social Assistance	137	11.8	213	12.0
Other Industry	530	45.4	682	38.3

* Slight differences in the column totals are due to rounding.

**Table 2:** Leave Status by Business Size for Respondents Working Full or Part-time Outside the Home (Fall 2020) and Full or Part-time, Inside or Outside the Home (Fall of 2021).

	Business Size (Number of Employees)							
	1–10		11–50		51–100		>100	
	*n*	% Yes	*n*	% Yes	*n*	% Yes	*n*	% Yes
	Fall 2020							
Paid leave if I have COVID-19 symptoms	44 _a_	17	57 _a_	21	67 _b_	44	195 _b_	40
Paid leave if I test positive for COVID-19	45 _a_	18	69 _a_	26	64 _b_	42	238 _b_	49
Unpaid leave—time off without pay	73 _a_	28	63 _a,b_	23	20 _b_	13	92 _b_	19
No leave—I cannot take off	35 _a_	14	24 _a_	9	9 _a,b_	6	21 _b_	4
Do not know	92 _a_	36	96 _a_	35	34 _b_	23	95 _b_	20
	Fall 2021							
General paid annual/vacation leave	124 _a_	35	205 _b_	61	120 _b_	72	794 _c_	87
General paid sick leave	97 _a_	27	183 _b_	55	107 _b,c_	65	667 _c_	73
Paid leave only for COVID-19 symptoms	23 _a_	6	34 _a,b_	10	27 _b,c_	16	169 _c_	19
Paid leave only for positive COVID-19 test	21 _a_	6	43 _b_	13	33 _b,c_	20	226 _c_	25
Unpaid leave—time off without pay	125 _a_	35	108 _a_	32	48 _a_	29	346 _a_	38
No leave—I cannot take off	53 _a_	15	11 _b_	3	4 _b_	3	15 _b_	2
Do not know	58 _a_	16	39 _a_	12	17 _a_	10	43 _b_	5

Note: Values in the same row not sharing the same subscript (a, b, or c) are significantly different (*p* < 0.05) in the two-sided test of equality for column proportions. Tests assume equal variances.

## Data Availability

Not applicable.

## Supplementary Materials

The following are available online at https://www.mdpi.com/article/10.3390/ijerph20042894/s1, Table S1: Self-reported mitigation strategies implemented by business size for respondents working outside the home in fall 2020 and fall 2021; Table S2: Differences in COVID-19 mitigation strategies by industry, fall 2020; Table S3: Differences in COVID-19 mitigation strategies by industry, fall 2021.
