# Supplementary material for: COVID-19 Workplace Mitigation Strategies and Employee Leave Policies Implemented during the Height of the Pandemic, United States, Fall 2020 and 2021
Source: Int J Environ Res Public Health. 2023 Feb 7;20(4):2894. doi: 10.3390/ijerph20042894 (PMC9956394; doi:10.3390/ijerph20042894)
Supplement: Supplementary file 1 [file ijerph-20-02894-s001.zip › ijerph-2060098-supplementary.pdf]

Supplementary Materials

Table S1. Self-reported Mitigation Strategies Implemented by Business Size for Respondents Working Outside the Home in Fall 2020 and Fall 2021

	Fall 2020							
	1–10		11–50		51–100		>100	
	n	% Yes	*n*	% Yes	*n*	% Yes	*n*	% Yes
Implemented safe distancing (6 feet or more) between employees and/or customers	124_{a}	48	163_{b}	60	98_{b}	64	367_{c}	76
Provided respirators (like N95s) to employees*	20_{a}	8	$30_{a,b}$	11	$29_{b,c}$	19	124_{c}	26
Required employees to wear a mask	126_{a}	49	200_{b}	74	108_{b}	71	420_{c}	86
Required customers/clients to wear masks	133_{a}	52	157_{a}	58	$91_{a,b}$	60	340_{b}	70
Screened employees (like asking about symptoms, taking temperatures, etc.)	74_{a}	29	128_{b}	47	$78_{b,c}$	51	301_{c}	62
Screened customers/clients (like asking about symptoms, taking temperatures, etc.)	63_{a}	25	73_{a}	27	$56_{a,b}$	37	204_{b}	42
Reassigned workers at increased risk for severe illness (older, underlying conditions)	15_{a}	6	23_{a}	8	28_{b}	19	124_{b}	26
Put up physical barriers like partitions or sneeze guards	64_{a}	25	84_{a}	31	49_{a}	32	245_{b}	50
Used enhanced cleaning/disinfection procedures	127_{a}	49	159_{a}	59	93_{a}	61	362_{b}	75
Tested employees for the virus that causes COVID-19	22_{a}	9	23_{a}	8	14_{a}	9	114_{b}	24
Provided COVID-19 prevention training to employees	57_{a}	22	$72_{a,b}$	27	$59_{b,c}$	39	244_{c}	50
Limited the number of customers in the establishment at one time	$108_{a,b}$	42	96_{a}	35	$69_{a,b}$	45	226_{b}	47
Provided hand sanitizer or handwashing supplies (like soap, drying materials)	162_{a}	63	202_{b}	75	$114_{a,b}$	75	414_{c}	85
Posted signs about safe practices (like social distancing, masks, handwashing)*	125_{a}	49	184_{b}	68	103_{b}	68	402_{c}	83
None of these actions taken to prevent spread of COVID-19	51_{a}	20	14_{b}	5	11_{b}	7	17_{b}	3
	Fall 2021							
Implemented safe distancing (6 feet or more) between employees and/or customers	97_{a}	27	132_{b}	39	70_{b}	41	513_{c}	56
Required employees to wear a mask	101_{a}	28	190_{b}	57	105_{b}	63	684_{c}	74
Required customers/clients to wear masks	81_{a}	22	140_{b}	42	73_{b}	43	503_{c}	55
Screened employees or customers (like asking about symptoms, taking temperatures)†	38_{a}	11	79_{b}	24	$48_{b,c}$	28	353_{c}	38
Required employees to be vaccinated against COVID-19§	28_{a}	8	58_{b}	17	$31_{b,c}$	19	260_{c}	28
Allowed reassignment of workers at increased risk	12_{a}	3	20_{a}	6	$11_{a,b}$	6	114_{b}	12
Put up physical barriers like partitions or sneeze guards	43_{a}	12	72_{b}	22	$39_{b,c}$	23	292_{c}	32
Used enhanced cleaning/disinfection procedures	94_{a}	26	139_{b}	42	$79_{b,c}$	47	505_{c}	55
Used enhanced ventilation at the worksite§	21_{a}	6	21_{a}	6	24_{b}	14	133_{b}	14
Testing employees for the virus that causes COVID-19	9_{a}	3	25_{b}	7	23_{b}	13	208_{c}	23
Provided COVID-19 prevention training to employees	30_{a}	8	64_{b}	19	29_{b}	17	279_{c}	30
Limited the number of customers in the establishment at one time	52_{a}	14	$61_{a,b}$	18	$42_{b,c}$	25	248_{c}	27
Provided hand sanitizer or handwashing supplies (like soap, drying materials)	138_{a}	38	208_{b}	62	$112_{b,c}$	66	646_{c}	70
Moved to remote working (such as teleworking)§	34_{a}	9	54_{b}	16	42_{b}	25	399_{c}	43
None of these actions taken to prevent spread of COVID-19	152_{a}	42	38_{b}	11	$14_{b,c}$	9	55_{c}	6

Note: Values in the same row not sharing the same subscript are significantly different at $p< .05$ in the two-sided test of equality for column proportions. Cells with no subscript are not included in the test. Tests assume equal variances.

* Item removed in fall 2021

† Combined item from fall 2020 and fall 2021

§ New item added in fall 2021

Table S2. Differences in COVID-19 Mitigation Strategies by Industry Fall 2021

	Manufacturing		Retail Trade		Information		Finance and Insurance		Professional, Scientific, and Technical Services		Educational Services		Health Care and Social Assistance		Other Industry	
	n	% Yes	*n*	% Yes	*n*	% Yes	*n*	% Yes	*n*	% Yes	*n*	% Yes	*n*	% Yes	*n*	% Yes
Paid leave if I have COVID-19 symptoms	$43_{a,b}$	34	24_{a}	19	$4_{a,b}$	29	21_{b}	49	$32_{b,c}$	43	$37_{a,b}$	34	$44_{a,b}$	32	$158_{a,b}$	30
Paid leave if I test positive for COVID-19	53_{a}	42	48_{a}	37	4_{a}	28	19_{a}	45	29_{a}	38	48_{a}	45	48_{a}	35	167_{a}	32
Unpaid leave - time off without pay	33_{a}	26	24_{a}	18	2_{a}	16	3_{a}	7	16_{a}	21	20_{a}	19	26_{a}	19	124_{a}	23
No leave - I cannot take off	9_{a}	7	14_{a}	11	0^{1}	0	3_{a}	6	3_{a}	4	4_{a}	3	5_{a}	4	53_{a}	10
Don't know	20_{a}	16	46_{b}	35	$6_{a,b}$	42	$8_{a,b}$	19	$16_{a,b}$	22	$32_{a,b}$	29	46_{b}	34	$142_{a,b}$	27
Implemented safe distancing (6 feet or more) between employees and/or customers	$85_{a,b}$	67	$93_{a,b}$	71	$8_{a,b}$	56	$29_{a,b}$	67	$52_{a,b}$	70	80_{a}	74	$98_{a,b}$	72	305_{b}	58
Provided respirators (like N95s) to employees	$32_{a,c,d}$	25	8_{b}	7	$1_{a,b,c}$	4	$2_{a,b}$	4	5_{b}	7	9_{b}	9	58_{c}	43	$88_{b,d}$	17
Required employees to wear a mask]	$94_{a,c,e}$	74	$103_{a,c,e}$	78	$8_{a,b}$	51	$34_{a,c,e}$	79	$58_{a,c,e}$	78	96_{c}	89	$120_{c,d}$	88	$342_{b,e}$	65
Required customers/clients to wear masks	74_{a}	58	$87_{a,b,c}$	66	5_{a}	35	$22_{a,b}$	52	$49_{a,b,c}$	66	$84_{b,c}$	78	109_{c}	80	291_{a}	55
Screened employees for symptoms	$85_{a,c}$	67	$56_{b,d}$	43	$4_{a,b,d}$	26	$15_{b,d}$	34	$37_{a,b,d}$	50	$66_{a,b,c}$	61	102_{c}	75	216_{d}	41
Screened customers/clients for symptoms	52_{a}	41	23_{b}	18	$1_{a,b}$	9	$10_{a,b}$	24	$26_{a,b}$	35	54_{a}	50	98_{c}	71	131_{b}	25
Reassigned workers at increased risk for severe illness	$19_{a,b}$	15	$16_{a,b}$	12	$1_{a,b}$	5	$7_{a,b}$	16	$15_{a,b}$	20	28_{a}	26	$31_{a,b}$	23	73_{b}	14
Put up physical barriers like partitions or sneeze guards	$49_{a,b,c}$	38	74_{a}	57	$3_{a,b,c}$	23	$16_{a,b,c}$	37	$20_{b,c}$	27	$54_{a,b}$	50	$67_{a,b}$	49	159_{c}	30
Used enhanced cleaning/disinfection procedures	$98_{a,c}$	77	$84_{a,b}$	64	5_{b}	31	$30_{a,b,c}$	72	$48_{a,b,c}$	64	90_{c}	84	$98_{a,c}$	71	$289_{b,d}$	55
Tested employees for the virus that causes COVID-19	18_{a}	14	7_{a}	6	$1_{a,b}$	5	2_{a}	4	8_{a}	11	10_{a}	9	50_{b}	36	79_{a}	15
Provided COVID-19 prevention training to employees	42_{a}	33	30_{a}	23	$4_{a,b}$	25	11_{a}	26	21_{a}	28	62_{b}	58	$82_{b,c}$	60	179_{a}	34
Limited the number of customers in the establishment at one time	$52_{a,b}$	41	$58_{a,b}$	44	$3_{a,b}$	21	$20_{a,b}$	47	$29_{a,b}$	40	$57_{a,b}$	53	74_{a}	54	206_{b}	39
Provided hand sanitizer or handwashing supplies	107_{a}	84	$95_{a,b}$	72	$11_{a,b}$	74	$38_{a,b}$	90	$65_{a,b}$	87	$90_{a,b}$	83	$113_{a,b}$	83	373_{b}	70
Posted signs about safe practices	$93_{a,b}$	73	$96_{a,b}$	73	$9_{a,b}$	58	$30_{a,b}$	70	$55_{a,b}$	74	91_{a}	84	109_{a}	80	332_{b}	63
None of these actions taken	3_{a}	2	$8_{a,b}$	7	$3_{a,b}$	18	$1_{a,b}$	3	$4_{a,b}$	5	$6_{a,b}$	6	4_{a}	3	64_{b}	12

Note: Values in the same row and subtable not sharing the same subscript are significantly different at $p< .05$ in the two-sided test of equality for column proportions.

Differences in COVID-19 Mitigation Strategies by Industry Fall 2021

	Manufacturing		Retail Trade		Information		Finance and Insurance		Professional, Scientific, and Technical Services		Educational Services		Health Care and Social Assistance		Other Industry	
	n	% Yes	*n*	% Yes	*n*	% Yes	*n*	% Yes	*n*	% Yes	*n*	% Yes	*n*	% Yes	*n*	% Yes
General paid annual/vacation leave	130_{a}	91	107_{b}	58	$51_{a,c}$	84	113_{a}	89	$161_{a,c}$	83	101_{b}	62	$153_{b,c}$	72	426_{b}	63
General paid sick leave	$91_{a,c,d}$	63	81_{b}	44	$44_{a,c,d}$	71	$96_{a,c}$	75	$120_{a,c,d}$	62	125_{a}	77	$130_{c,d}$	61	$368_{b,d}$	54
Paid leave only for COVID-19 symptoms	39_{a}	27	$25_{a,b}$	14	$5_{a,b}$	8	$18_{a,b}$	14	17_{b}	9	$28_{a,b}$	18	$20_{b,c}$	10	$100_{b,d}$	15
Paid leave only for positive COVID-19 test	44_{a}	31	$41_{a,b}$	22	$6_{b,c,d}$	10	$25_{a,b,c}$	20	19_{c}	10	$37_{a,d,e}$	23	$36_{a,b,c}$	17	$116_{b,c,e}$	17
Unpaid leave - time off without pay	$55_{a,b}$	38	82_{a}	45	$24_{a,b}$	39	$44_{a,b}$	34	$61_{a,b}$	31	45_{b}	28	$74_{a,b}$	35	$244_{a,b}$	36
No leave - I cannot take off	2_{a}	2	8_{a}	5	1_{a}	2	2_{a}	2	5_{a}	3	3_{a}	2	11_{a}	5	49_{a}	7
Don't know	8_{a}	6	26_{a}	14	5_{a}	8	5_{a}	4	12_{a}	6	14_{a}	9	17_{a}	8	70_{a}	10

Safe distancing (6 feet or more) between employees and/or customers	79_{a}	54	84_{a}	45	22_{a}	35	68_{a}	52	95_{a}	49	79_{a}	47	106_{a}	50	279_{a}	41
Requiring employees to wear a mask	$88_{a,c,d}$	61	$135_{a,b,e}$	73	$32_{a,c,d}$	52	$80_{a,c,d,e}$	62	106_{c}	54	$121_{b,d,e}$	73	165_{e}	77	$352_{c,f}$	52
Requiring customers/clients to wear a mask	$69_{a,e,g}$	48	$75_{a,e,g}$	40	$15_{a,b}$	25	$49_{a,c}$	38	$73_{a,d}$	37	$94_{e,f}$	57	143_{f}	67	$276_{b,c,d,g}$	41
Screening employees or customers for symptoms, taking temperatures, etc.	$63_{a,d}$	44	$62_{a,b,d}$	34	$13_{a,b,e}$	21	$32_{b,e}$	24	$48_{b,c,e}$	25	$48_{a,b,e}$	29	101_{d}	47	149_{e}	22
Testing employees for the virus that causes COVID-19	20_{a}	13	14_{a}	7	5_{a}	8	11_{a}	8	24_{a}	12	49_{b}	30	73_{b}	34	68_{a}	10
Requiring employees to be vaccinated against COVID-19	16_{a}	11	18_{a}	10	$19_{b,c,d,e}$	30	$17_{a,b}$	13	$47_{b,c,e}$	24	$56_{c,d}$	34	96_{d}	45	$109_{a,e}$	16
Allowing reassignment of workers at increased risk (e.g., older, underlying conditions)	$16_{a,c}$	11	2_{b}	1	$3_{a,b}$	6	$11_{a,c}$	9	$20_{a,c}$	10	25_{a}	15	36_{a}	17	$43_{b,c}$	6
Using physical barriers like partitions or sneeze guards	$40_{a,c,d,e}$	28	$70_{a,b}$	38	$12_{a,c,d,e}$	20	$33_{a,c,d,e}$	26	33_{c}	17	$63_{b,d}$	38	$70_{b,e}$	33	$124_{c,f}$	18
Using enhanced cleaning/disinfection procedures	$74_{a,c,d,e}$	51	$97_{a,b,f}$	52	$26_{a,c,d,e}$	43	$57_{a,c,d,e}$	44	71_{c}	36	$92_{b,d}$	55	$129_{b,e}$	61	$271_{c,f}$	40
Using enhanced ventilation at the worksite	$12_{a,c}$	8	$14_{a,c}$	8	$7_{a,b,c}$	11	$14_{a,b,c}$	11	$22_{a,b,c}$	11	37_{b}	22	$36_{a,b}$	17	58_{c}	9
Providing COVID-19 prevention training to employees	$37_{a,c,d,e}$	26	$25_{a,b}$	14	$11_{a,c,e}$	18	$39_{c,d}$	30	$39_{a,c,e}$	20	$45_{a,c,d,e}$	27	87_{d}	41	$117_{b,e}$	17
Limiting the number of customers in the establishment at one time	$34_{a,d,e}$	24	$44_{a,d,e}$	24	$11_{a,d,e}$	18	$36_{a,d,e}$	28	$41_{a,b}$	21	$29_{a,c}$	18	77_{d}	36	$129_{b,c,e}$	19
Providing hand sanitizer or handwashing supplies (like soap, drying materials)	$100_{a,e,f}$	69	$118_{a,e,f}$	64	$30_{a,b}$	49	$73_{a,c}$	56	$111_{a,d}$	57	123_{e}	74	$148_{a,e,f}$	69	$399_{b,c,d,f}$	59
Moved to remote working (such as telework)	$35_{a,d}$	24	17_{b}	9	38_{c}	61	72_{c}	55	119_{c}	61	54_{a}	33	$54_{a,d}$	25	140_{d}	21
None of these actions taken to prevent spread of COVID-19	$23_{a,b}$	16	$26_{a,b}$	14	$10_{a,b}$	17	$17_{a,b}$	13	$24_{a,b}$	12	12_{a}	7	14_{a}	6	133_{b}	20

Note: Values in the same row and subtable not sharing the same subscript are significantly different at $p < .05$ in the two-sided test of equality for column proportions.
